# The pro-tumor effect of CD200 expression is not mimicked by agonistic CD200R antibodies

**DOI:** 10.1371/journal.pone.0210796

**Published:** 2019-01-17

**Authors:** Zofia Pilch, Katarzyna Tonecka, Marcin Skorzynski, Zuzanna Sas, Agata Braniewska, Tomasz Kryczka, Louis Boon, Jakub Golab, Linde Meyaard, Tomasz P. Rygiel

**Affiliations:** 1 Department of Immunology, Medical University of Warsaw, Warsaw, Poland; 2 Department of Experimental Pharmacology, Mossakowski Medical Research Centre Polish Academy of Sciences, Warsaw, Poland; 3 Bioceros BV, Utrecht, The Netherlands; 4 Centre for Preclinical Research and Technology, Medical University of Warsaw, Warsaw, Poland; 5 Laboratory of Translational Immunology, Department of Immunology, University Medical Center Utrecht, Utrecht, The Netherlands; 6 Oncode Institute, University Medical Center Utrecht, Utrecht, The Netherlands; Rutgers University, UNITED STATES

## Abstract

Tumor-infiltrating immune cells can impact tumor growth and progression. The inhibitory CD200 receptor (CD200R) suppresses the activation of myeloid cells and lack of this pathway results in a reduction of tumor growth, conversely a tumorigenic effect of CD200R triggering was also described. Here we investigated the role of CD200R activation in syngeneic mouse tumor models. We showed that agonistic CD200R antibody reached tumors, but had no significant impact on tumor growth and minor effect on infiltration of immune myeloid cells. These effects were reproduced using two different anti-CD200R clones. In contrast, we showed that CD200-deficiency did decrease melanoma tumor burden. The presence of either endogenous or tumor-expressed CD200 restored the growth of metastatic melanoma foci. On the basis of these findings, we conclude that blockade of the endogenous ligand CD200 prevented the tumorigenic effect of CD200R-expressing myeloid cells in the tumor microenvironment, whereas agonistic anti-CD200R has no effect on tumor development.

## Introduction

CD200 and its receptor (CD200R) are involved in the regulation of inflammation in various pathologies including autoimmune diseases, infections and cancer [1–4]. Lack of this regulatory pathway in *Cd200*^*–/–*^mice leads to exacerbation of autoimmune diseases e.g. autoimmune encephalomyelitis and collagen-induced arthritis in mice [2]. Immune checkpoints, such as the inhibitory CD200 receptor (CD200R) axis, play a dual role in balancing the immune system during microbial infection [3,5,6]. Moreover, several viruses e.g. human herpesvirus 8 or myxoma virus have CD200 orthologues that can trigger endogenous CD200R [7,8] and regulate myeloid cell function [9]. We previously showed, that lack of CD200R signaling strongly enhances type I interferon production and viral clearance, and improves the outcome of mouse hepatitis coronavirus infection, particularly in female mice, because CD200R ligation inhibits TLR7 signaling [10].

The tumor microenvironment is dominated by immune cells that play a key role in tumor progression and foster proliferation, survival, and migration of transformed cells [11–13]. On the other hand, immune cells in the tumor microenvironment could facilitate an anti-tumor cellular response if activated properly. Elevated levels of CD200 are found in multiple types of cancer and many reports suggest that it is responsible for the suppression of anti-tumor immune responses [14,15]. It was proposed that treatment with blocking anti-CD200 antibodies might be beneficial for patients with CD200-expressing cancers [16]. In addition, we previously showed that the absence of CD200R signaling pathway inhibits the growth of endogenous cancer cells even without CD200 expression in the tumor cells [17]. However, CD200 seems to have a dual role in cancer development and metastasis [4]. In the highly aggressive breast cancer 4THM animal model, the use of CD200R agonists exert a potent inhibitory effect on inflammation-driven carcinogenesis and metastasis [18]. In contrast, CD200-positive squamous cells carcinoma has an enhanced ability to metastasize [19]. Moreover, in the EMT6 breast carcinoma animal model CD200 overexpression results in increased lymph node metastasis [20]. These finding support tumorigenic effects of CD200R signaling in the tumor microenvironment in certain circumstances.

The *Cd200R* gene has undergone a rapid evolution in mice, creating a variety of inhibitory and activating receptors in different mice strains. Most common laboratory mouse strains (e.g. C57BL/6, Balb/c, C3H/Hej) have only one inhibitory CD200R (CD200R1), whereas CD200R2 is expressed in AKR, CD1 and NOD strains [21]. There are four paired activating receptors CD200RLa, CD200RLb, CD200RLc, and CD200RLe, only first of these is expressed by the C57BL/6, Balb/c, C3H/Hej mouse strains. This variety of CD200R alleles is not recapitulated in humans, since people have only one inhibitory and activating CD200R. In both mice and humans, the activating receptors do not bind CD200 and their natural ligand is unknown [22,23].

The specificity of agonistic monoclonal antibodies for these receptors was described in detail by Akkaya et al. [21]. The most widely used anti-mouse CD200R antibody—OX110, triggers the inhibitory receptor CD200R1 and activating receptor CD200RLc, whereas clone OX131, triggers CD200R1, CD200R2, and CD200RL. Since CD200R2 and CD200RL are not expressed in C57BL/6, Balb/c, C3H/Hej mouse strains, OX131 can be used as inhibitory CD200R1-specific in these strains.

Upon different stimulation, macrophages are able to differentiate into various subtypes as instructed by the tissue microenvironment. Traditionally macrophages are divided into two opposite phenotypes: classically and alternatively activated cells, M1, and M2 respectively [24]. Classical activation of macrophages is inhibited by CD200 which is expressed on cells in diverse tissues [2]. Koning et al. demonstrated that CD200R is expressed in human and mouse M2 cells [25]. Thus, triggering of CD200R provides an immunosuppressive signal and would contribute to the immune-regulatory capacity of M2 macrophages. However, this strategy may also repress pathways associated with classical macrophage activation.

In this study, we examined the role of the CD200-CD200R pathway in tumor development. Treatment with agonistic anti-CD200R did not inhibit tumor growth in several tumor models in mice with endogenous expression of CD200, indicating that further CD200R stimulation does not affect tumor growth. Thus, we investigated the effects of CD200 deficiency on the systemic development of mouse melanoma B16F10 cells. We showed that expression of CD200 on either host or tumor cells increased tumor burden. These results show that when CD200R considering as a therapeutic target, rather antagonistic than agonistic antibodies should be used.

## Materials and methods

### Mice and cell lines

Female wild type (WT) Balb/c mice were purchased from the Animal House of the Polish Academy of Sciences, Mossakowski Medical Research Center (Warsaw, Poland). Wild type and *Cd200*^-/-^ C57BL/6 mice were obtained from University Medical Center Utrecht and reared in the Animal House of the Maria Sklodowska-Curie Institute of Oncology (Warsaw, Poland). Experiments were performed at the Animal Facility of Medical University of Warsaw. At the end of all experiments, mice were sacrificed by anesthesia overdose. During the study, none of the mice experienced unintended discomfort requiring intervention to alleviate the suffering. All *in vivo* experiments and specific procedures and protocols used for this study were performed in accordance with the guidelines and approved by the First Local Ethics Committee for the Animal Experimentation in Warsaw. Mice with tumors that did not have typical shape or the size, before the start of the therapy, were excluded from the experiment. The murine breast mammary carcinoma EMT6, Lewis lung carcinoma LLC and B16F10 melanoma cell lines were purchased from American Type Culture Collection (Manassas, VA, USA) and were maintained in RPMI 1640 medium supplemented with 10% (v/v) heat-inactivated fetal calf serum (FCS), (Hyclone) and antibiotic/antimycotic solution (Sigma). B16F10-EV and B16F10-CD200 melanoma cells with stable expression of Firefly luciferase were generated by transduction with pMX-luc/neo expression vector with or without full-length mouse CD200 and cultured under neomycin selection. B16F10 melanoma cells were maintained in DMEM supplemented with 10% (FCS) and penicillin/streptomycin mixture (Sigma).

### Tumor models

Superficial tumors models were induced in Balb/c or C57BL/6 mice by subcutaneous inoculation of cells into the right thigh on day 0. EMT6, LLC (1.5–3 × 10^5^) cells were injected in 30 μl of PBS: Matrigel Growth Factor Reduced (Corning, LifeSciences, USA) mixture (1:1). Mice were injected into a tail vein once every 3 days, starting from day 6–7, with anti-CD200R (clone OX110 or OX131) or matching control antibody GL117 or rat anti-IgG1 (RTK2071) respectively, 100 μg per dose. Tumor was measured with caliper and the volume was calculated according to the formula: [mm^3^] = (length [mm]) × (width [mm])^2^/2. Growth of flat-shaped tumors was calculated as their surface, according to the formula: [mm^2^] = (length [mm]) × (width [mm])/2.

B16F10 melanoma with or without stable expression of CD200, together with luciferase marker were used (B16F10-EV and B16F10-CD200 respectively). *Cd200*^-/-^ mice and wild type controls were injected on the right flank with a mixture of B16F10 cells (1.5 × 10^5^) in 30 μl PBS/Matrigel mixture. Melanoma tumor growth was monitored by *in vivo* bioluminescence imaging. To that end, mice were injected (*i*.*p*.) with D-luciferin (Syd Labs, Natick, MA, USA) (150 mg/kg), and after 5 min were anesthetized with isoflurane and visualized using the IVIS Imaging System (Xenogen, Alameda, CA, USA). Images were analyzed with the Living Image 4.3 software package (Caliper Life Science, Hopkinton, MA, USA). To quantify the bioluminescence (BLI) signal of mice, the regions of interest (ROIs) were drawn on the tumor region and results were used to generate growth curves of B16F10 melanoma. Bioluminescence data were presented as average radiance (photons/sec/cm^2^/steradian).

### Flow cytometry

Tumors were cut into small pieces, digested for 30 min at 37°C using Collagenase type IV (600U) (Sigma) and (400U) DNAse (Sigma). Next, tissue fragments were dissociated using a gentleMACS Dissociator, and filtered through a 100 μm cell strainer, washed with PBS containing 2 mM EDTA and 1% FCS, centrifuged and stained. Spleens were forced through the cell strainer (70 μm) and cells were centrifuged (500g) at 4ºC. When necessary, erythrocytes were lysed using a buffer containing 155 mM NH_4_Cl, 10 mM NaH_2_CO_3,_ and 0.1 mM EDTA, pH 7.3. For staining, cells were blocked in 5% normal rat serum and stained with fluorescently labeled monoclonal antibodies: anti-CD11b (M1/70, 53-0112-82), anti-Ly6C (HK1.4, 17-5932-80), anti-CD200R (OX110, 12-5201-82), anti-MHC-II (M5/114.15.2, 25-5321-80) (eBioscicence, USA), anti-F4/80 (BM8, 123118) (BioLegend), anti-TNF-α (XT22, 554419), anti-IFN-γ (XMG1.2, 563376) (BD Pharmingen). For intracellular staining of TNF-α or IFN-γ, cells were first stimulated with PMA (LC Laboratories) / ionomycin (Thermo Fisher), and GolgiStop (BD Pharmingen) for at least 5 hrs and subsequently stained using Intracellular Fixation and Permeabilization Buffer Set (eBioscience) according to the manufacturer’s instructions.

### Statistics

Data were analyzed using GraphPad Prism 7.03 software. Statistical significance was calculated using Student's-t test or Mann-Whitney test when appropriate.

## Results and discussion

**Fig 1:**
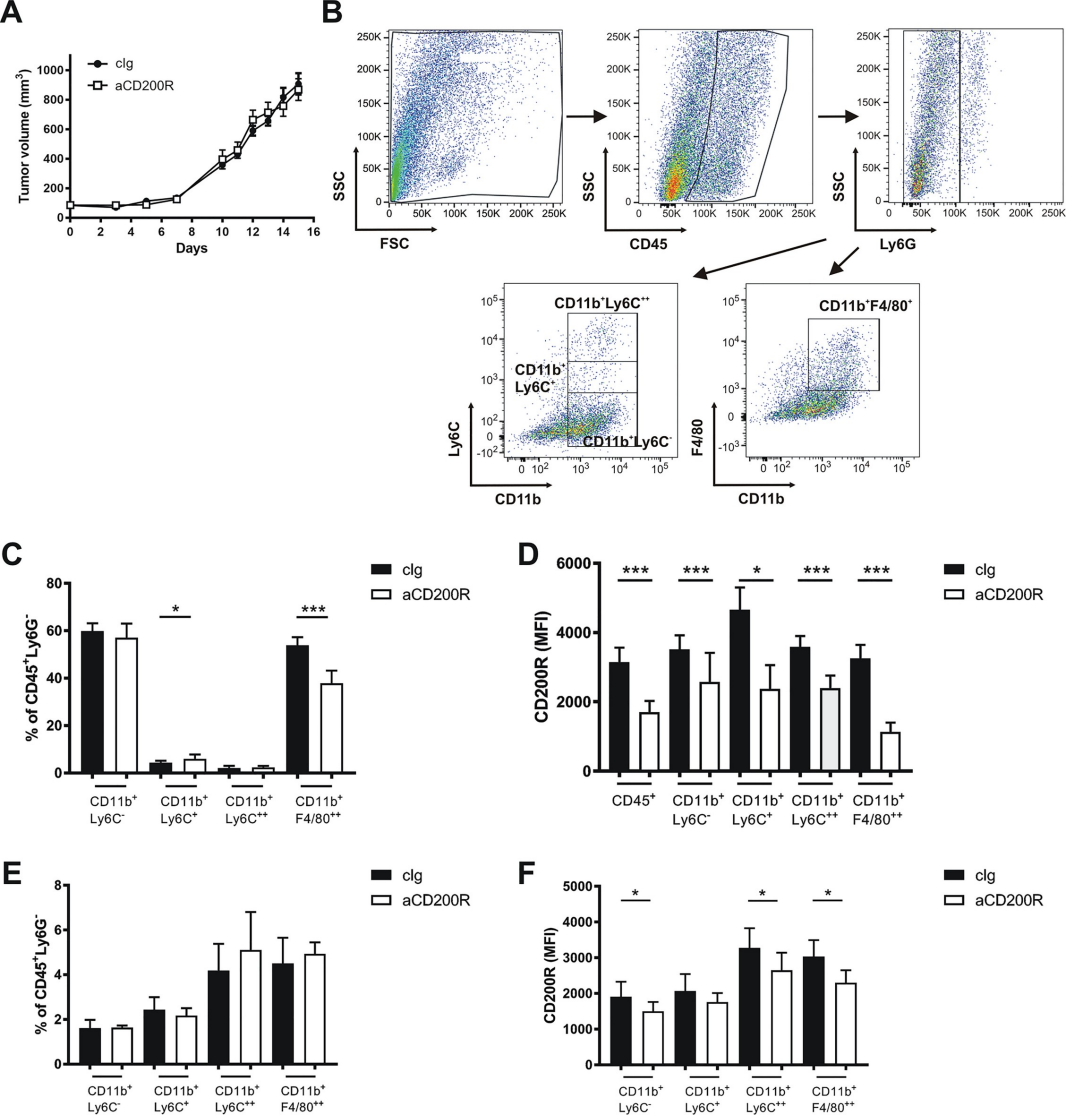
Impact of anti-CD200R antibodies (clone OX110) on the growth of EMT6 tumors. (A) EMT6 tumor growth measured as a tumor volume. (B) Example of the gating strategy in flow cytometric analysis of the intratumoral immune cells. (C) Percentage of intratumoral myeloid cells (CD11b^+^) without neutrophils (Ly6G^+^). (D) Expression of CD200R in intratumoral immune cells. (E) Percentage of splenic myeloid cells from mice with EMT6 tumors, gating as in the tumors. (F) Expression of CD200R in splenic immune cells. Mice were treated with control or anti-CD200R antibodies (clone OX110). N = 7–8 / group, data are presented as mean ± SEM. *P < 0.05, **P < 0.01, ***P < 0.001.

To investigate the role of therapeutic modulation of the CD200-CD200R signaling pathway in tumor progression, mice subcutaneously inoculated with mammary gland cancer cells (EMT6) and were treated with agonistic anti-CD200R (clone OX110) or isotype control. Antibodies were administered 3–5 times *i*.*v*. at a dose of 100 μg every third day, starting from day 7 after inoculation of tumor cells. Treatment with agonistic anti-CD200R had no significant effect on the growth of EMT6 tumors (Fig 1A). We analyzed tumor-infiltrating immune myeloid cells according to the gating strategy presented in Fig 1B. Upon anti-CD200R treatment, we found a decrease in the fraction of tumor macrophages (CD11b^+^F4/80^+^) and a simultaneous but smaller, increase in the percentage of CD11b^+^Ly6C^+^ cells (Fig 1C). Intratumoral monocytes (CD11b^+^Ly6C^++^) and remaining myeloid cells (CD11b^+^Ly6C^-^) were not affected by the CD200R agonist (Fig 1C). Anti-CD200R treatment decreased fluorescence of CD200R by flow cytometry using the same antibody clone (OX110) for the detection (Fig 1D), in all analyzed intratumoral immune cell populations, proving tumor penetration by intravenously administered anti-CD200R. Similarly, we analyzed the composition of splenic myeloid cell populations. We found no significant differences in the frequency of these cells (Fig 1E). CD200R expression was significantly decreased in all analyzed populations with the exception of CD11b^+^Ly6C^+^ cells (Fig 1F). However, relative differences observed in splenocytes were smaller than in the tumor subpopulations.

**Fig 2:**
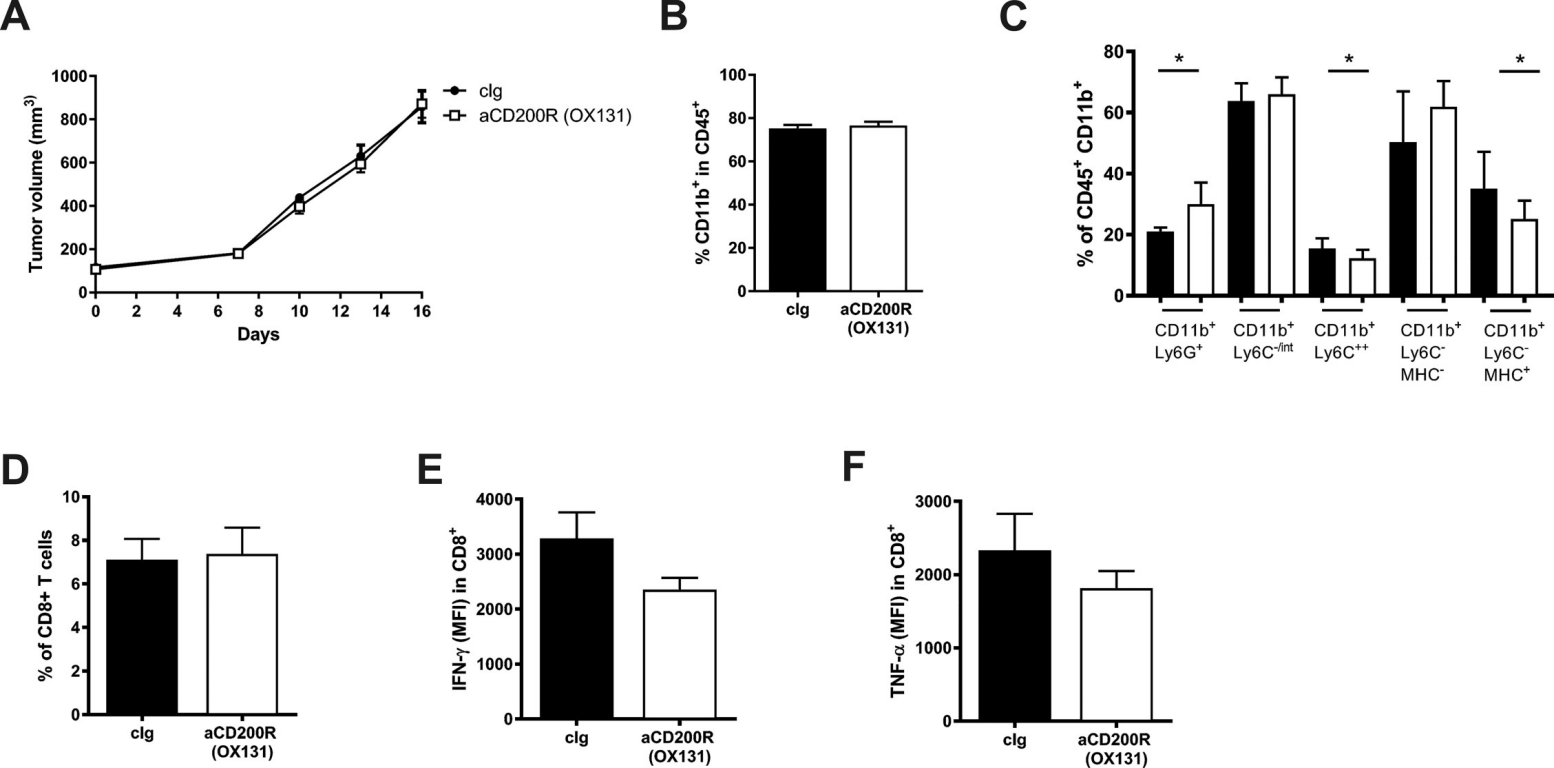
Impact of anti-CD200R antibodies (clone OX131) on the growth of EMT6 tumors. (A) EMT6 tumor growth measured as a tumor volume. (B) Percentage of intratumoral myeloid cells (in immune gate CD45^+^). (C) Percentage of intratumoral myeloid cells—subpopulations in CD11b^+^ cells. (D) Percentage of intratumoral CD8^+^ T lymphocytes. Expression of IFN-γ and TNF-α in the intratumoral CD8^+^ T lymphocytes (E) (F) respectively. Mice were treated with control or anti-CD200R (clone OX131). N = 7–10 / group, data are presented as mean ± SEM. *P < 0.05.

To exclude the possibility that the lack of therapeutic effect of the agonistic antibody (OX110) was due to simultaneous triggering of inhibitory (CD200R1) and activating (CD200RLc) receptors, we conducted an experiment with an anti-CD200R1 agonistic antibody (clone OX131) that, in Balb/c mice, does not trigger CD200RLc [21]. Similarly to OX110, intravenous administration of OX131 did not affect the growth of EMT6 tumors (Fig 2A). Cytometric analysis of intratumoral immune cells showed no effect of the OX131 clone on the myeloid cell infiltration (Fig 2B), similar to OX110 (Fig 1C).

More detailed analysis showed several significant differences in the intratumoral cell populations upon anti-CD200R1 treatment. The fraction of granulocytic myeloid cells (CD11b^+^Ly6G^+^) was increased after OX131 treatment (Fig 2B). In contrast, the monocytic myeloid cells (CD11b^+^Ly6C^+^) and intratumoral macrophages (CD11b^+^Ly6C^-^MHC-II^+^) were decreased (Fig 2C). These results were similar to the treatment with OX110 where intratumoral macrophages (CD11b^+^F4/80^+^) were also significantly decreased (Fig 1C). The fraction of intratumoral CD8^+^ T cells was not different (Fig 2D). To measure the activity of intratumoral lymphocytes, cells were stimulated *ex vivo* with PMA/ionomycin and stained for the intracellular cytokine production. We found decreased IFN-γ (Fig 2E) and TNF-α (Fig 2F) in tumor-infiltrating CD8^+^ T cells, but these differences were not statistically significant. These data suggest that even with the use of anti-CD200R (clone OX131) that is specific for the inhibitory CD200R1, there is no significant effect of anti-CD200R on tumor growth or the composition of tumor-infiltrating immune cells.

**Fig 3:**
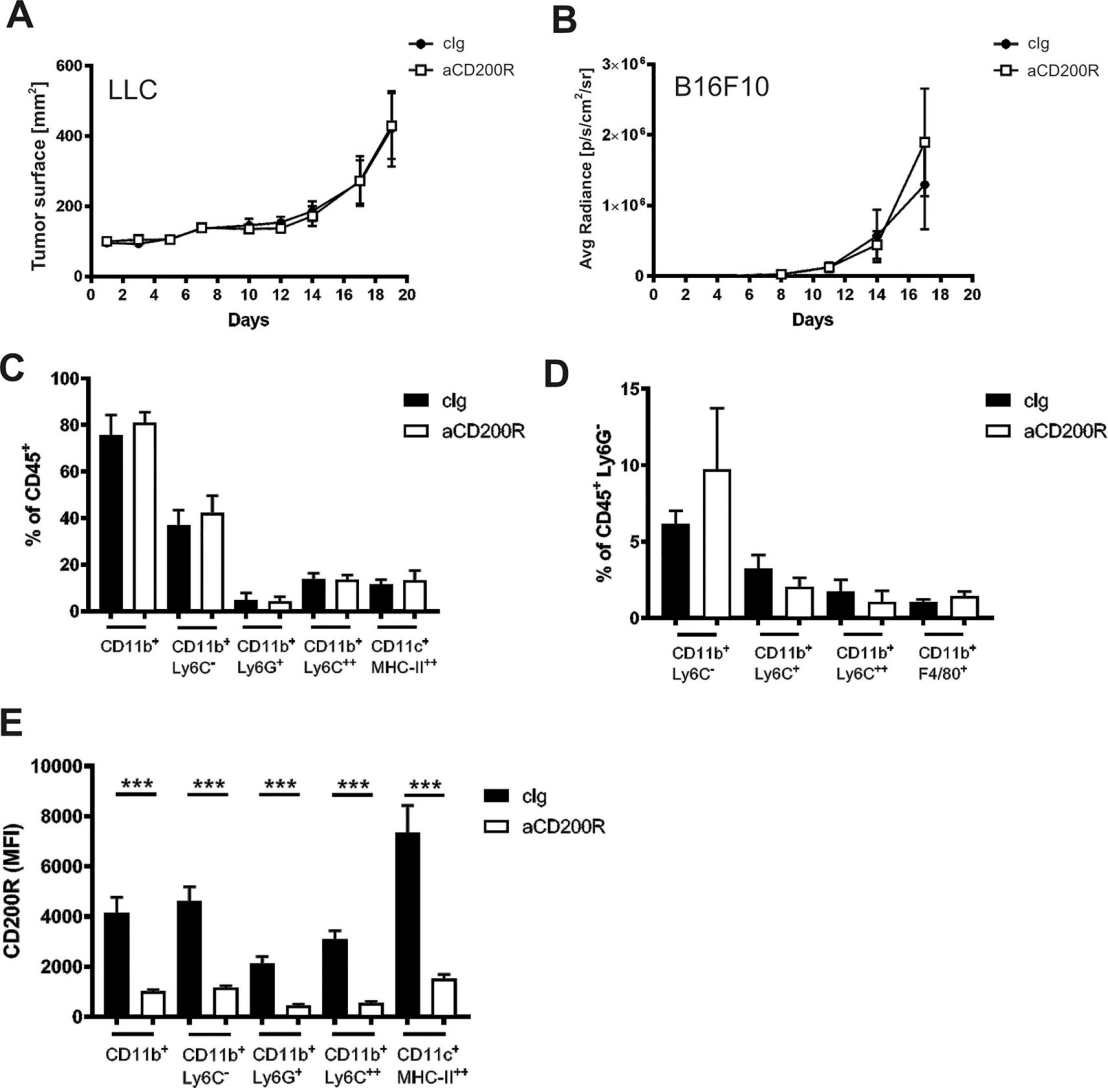
Impact of anti-CD200R antibodies on the growth of LLC and B16F10 tumors. (A) Tumor growth depicted as tumor surface of LLC, N = 5 / group. (B) Tumor burden quantified as luminescence of the luciferase expressed by tumor cells in lungs (B16F10), N = 10 / group. (C) Percentage of intratumoral myeloid cells (CD11b^+^) in LLC. (D) Percentage of myeloid cells in lungs with B16F10 tumor foci. (E) Expression of CD200R in intratumoral immune cells of LLC tumors. Mice were treated with control or anti-CD200R (clone OX110). Data are presented as mean ± SEM. *P < 0.05, **P < 0.01, ***P < 0.001.

To analyze the potential therapeutic effect in more immunogenic models, we analyzed the effect of anti-CD200R (OX110) treatment in two distinct tumor models: LLC (Lewis lung carcinoma) and B16F10 (melanoma). These tumor models are known to be more immunogenic and are syngeneic to the C57BL/6 mice strain that has more proinflammatory characteristics. Again we found no significant effect of anti-CD200R on the growth of subcutaneously inoculated LLC (Fig 3A) nor of intravenously inoculated B16F10, resulting mostly in foci formation in the lungs (Fig 3B). Since LLC grew in flat tumors we decided to quantify its growth as a surface, whereas B16F10 growth was monitored by light emitted from luciferase, expressed by tumor cells. Again in LLC tumors, we did not observe any difference in the percentage of main myeloid cell populations (Fig 3C). Similarly, in the lungs of B16F10 tumor-bearing mice, no difference in the percentage of main myeloid cell populations was found (Fig 3D). We investigated the expression of CD200R and observed a significant decrease of CD200R on intratumoral myeloid cells upon the intravenous treatment with anti-CD200R (Fig 3E), proving penetration of antibody into the subcutaneous LLC tumor. These results suggest that agonistic anti-CD200R has no effect on tumor growth in wild-type mice with endogenous CD200 ligand.

**Fig 4:**
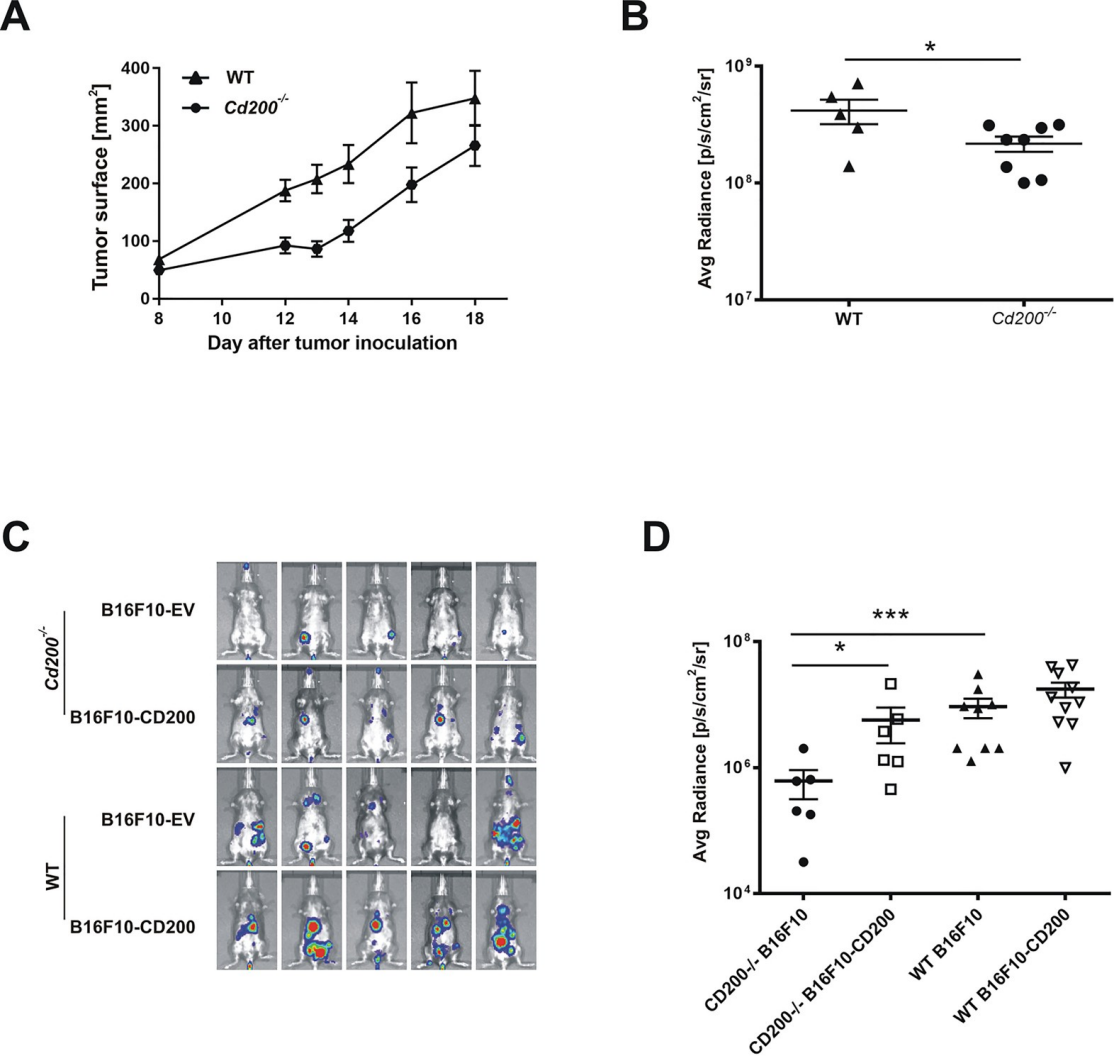
Impact of CD200-CD200R signaling on the growth of subcutaneous B16F10 melanoma. (A) Tumor (B16F10) growth expressed as a tumor surface measured by caliper in *Cd200*^*-/-*^ and control (WT) mice inoculated subcutaneously, N = 5–8 / group. (B) Bioluminescence intensity from each of the tumors shown in (A) on day 18 after tumor cells inoculation. (C) Total body bioluminescence of mice inoculated intravenously with B16F10 cells. (D) Bioluminescence from chest areas of mice shown in (C) on day 20 after tumor cells inoculation, N = 6–10 / group. Data are presented as mean ± SEM, significance was calculated with *t*-test (A,B) or Mann-Whitney test (D) *P < 0.05, ***P < 0.001.

To further investigate the role the CD200-CD200R axis on tumor growth we used luciferase-expressing B16F10 melanoma cells inoculated into syngeneic *Cd200*^*-/-*^ and control wild-type (WT) C57BL/6 mice. Both, direct measurements of tumor volume (Fig 4A), as well as bioluminescence (BLI) measurements of the same tumors (Fig 4B), revealed that *Cd200*^*-/-*^ mice developed smaller tumors as compared to control WT mice (Fig 4A). B16F10 cells with (B16F10-CD200 cells) or without (B16F10-EV cells) CD200 expression were inoculated *i*.*v*. into WT or *Cd200*^*-/-*^ mice to see whether tumor-expressed CD200 can affect tumor growth. Intravenously inoculated B16F10 cells disseminate to the lungs and other organs and their growth can be monitored with BLI. Regardless of the CD200 expression in tumor cells, control WT mice had higher tumor burden as compared to *Cd200*^*-/-*^ mice (Fig 4C and 4D), similarly to the subcutaneous tumor models (Fig 4A and 4B). Although the lung tumor burdens after inoculation of B16F10-CD200 and B16F10-EV cells was comparable in WT mice, the CD200 negative tumors grew significantly slower in the lungs of *Cd200*^*-/-*^ mice as compared to CD200 expressing melanomas (Fig 4C and 4D). These results indicate that tumor development is accelerated by both endogenous and tumor-derived CD200 expression. CD200R-signaling affects the growth and progression of cancer, however, its precise mechanism of action is not clear. Lack of CD200 expression inhibits endogenous skin tumors development [17]. Additionally, expression of CD200 on cancer cells correlates with poor patient prognosis [14] and increases the frequency of immunosuppressive Treg cells [20]. All these indicate a positive effect of CD200R on tumor growth by inhibition of the antitumor response. However, a tumor-supportive effect is also possible when CD200R signaling inhibits the activity of pro-tumor myeloid cells [19,26]. The dominant effect probably depends on tumor immunogenicity, composition of myeloid cells expressing the CD200R and the tumor inflammation.

Anti-CD200R diminishes production of cytokines by CD11b^+^ myeloid cells, indicating that the antibody suppresses function of myeloid cells by engaging CD200R [26]. Triggering of CD200R by agonistic anti-CD200R reduces CD200-negative melanoma tumor formation in the lungs particularly via inhibition of a Gr1^+^ myeloid cell population, that feasibly protects metastatic cancer cells from surveillance by immune cells [26]. Importantly, cancer cells used in that study overexpressed ovalbumin, a strong adjuvant able to induce an adoptive anticancer response. In our study, injection of agonistic anti-CD200R antibodies as a monotherapy did not affect the growth of CD200-negative subcutaneous tumors: melanoma, breast, and lung carcinoma in syngeneic tumor models. This suggests that in the absence of immunogenic antigen, e.g. OVA, agonistic anti-CD200R is not effective in the presence of endogenous CD200. Using CD200-positive and CD200-negative melanoma tumor cells, we showed that lack of CD200-CD200R interaction in a *Cd200*^*-/-*^ host does inhibit tumor growth of CD200-negative cells. Expression of CD200 either on host or melanoma cells promotes tumor cell growth. However, triggering of CD200R by agonistic anti-CD200R did not further enhance tumor growth.

In this study, using several additional tumor models with various degree of intratumoral inflammation, we confirmed that anti-CD200R mono treatment does not have therapeutic effect without additional inflammation. In contrast, using a model of colon carcinoma we have recently shown that anti-CD200R treatment suppresses tumor growth and modifies immune cell recruitment but only in the presence of TLR7-induced inflammation [27].

## Conclusions

Modulation of CD200R signaling might be important for the treatment of cancer. Treatment with agonistic anti-CD200R has no effect on tumor development without additional immune/inflammatory stimulation. However, the blockade of CD200-CD200R interaction could inhibit tumor growth, supporting antagonistic CD200 or CD200R antibodies as a treatment option in cancer. In this case, expression of CD200R on intratumoral immune cells could be a positive indicator of the use of blocking anti-CD200R antibodies.

## Supporting information

S1 Datasets Tumor volume and flow cytometric data presented in the Fig 1. (ZIP)

S2 Datasets Tumor volume and flow cytometric data presented in the Fig 2. (ZIP)

S3 Datasets Tumor measurement data and flow cytometric data presented in the Fig 3. (ZIP)

S4 Datasets Tumor measurement data presented in the Fig 4. (ZIP)
